# Deep Convolutional Clustering-Based Time Series Anomaly Detection

**DOI:** 10.3390/s21165488

**Published:** 2021-08-15

**Authors:** Gavneet Singh Chadha, Intekhab Islam, Andreas Schwung, Steven X. Ding

**Affiliations:** 1 Department of Automation Technology, South Westphalia University of Applied Sciences, 59494 Soest, Germany; islam.intekhab@fh-swf.de (I.I.); schwung.andreas@fh-swf.de (A.S.); 2 Department of Automatic Control and Complex Systems, University of Duisburg-Essen, 47057 Duisburg, Germany; steven.ding@uni-due.de

**Keywords:** unsupervised learning, deep convolutional autoencoder, top-K K-means clustering, anomaly detection

## Abstract

This paper presents a novel approach for anomaly detection in industrial processes. The system solely relies on unlabeled data and employs a 1D-convolutional neural network-based deep autoencoder architecture. As a core novelty, we split the autoencoder latent space in discriminative and reconstructive latent features and introduce an auxiliary loss based on k-means clustering for the discriminatory latent variables. We employ a Top-K clustering objective for separating the latent space, selecting the most discriminative features from the latent space. We use the approach to the benchmark Tennessee Eastman data set to prove its applicability. We provide different ablation studies and analyze the method concerning various downstream tasks, including anomaly detection, binary and multi-class classification. The obtained results show the potential of the approach to improve downstream tasks compared to standard autoencoder architectures.

## 1. Introduction

Sophisticated and interconnected modern manufacturing systems require transparent and insightful analytics. Consequently, intelligent condition monitoring of such processes is necessary to analyze changes in the process parameters and determine anomalies that hurt the reliability of the overall system. This unreliability can also lead to substantial financial consequences. However, modern production systems constitute complex interconnected behaviour, which renders the derivation of models through the first principle very difficult [1]. Hence, data-driven methods are an appealing alternative, particularly as a huge amount of data ranging from field level devices like sensors and actuators to manufacturing execution systems and enterprise resource planning systems are available through the Industrial Internet of Things [2].

However, a significant part of data-driven methods, namely supervised machine learning relies on the availability of labelled data from all of the possible operating conditions of the system. This availability of labelled data for industrial processes is infeasible due to various reasons. First, the faulty or abnormal operation often results in shutdowns or instantaneous repair actions, such that sufficient data instances are lacking. Second, data set labelling has to be done manually, which is usually not accomplished in industrial practice. Third, data sets for inconceivable fault cases are impossible to gather. In such cases, unsupervised or semi-supervised learning based data-driven techniques is the only alternative as they can suitably characterize the fault-free state of the system, which can subsequently be used to assess abnormal or faulty conditions.

Unsupervised or semi-supervised methods have been aggressively used in the area of novelty or anomaly detection. As surveyed in [3], the methods for anomaly detection can be categorized into Probabilistic Models, Distance-based Models, Reconstruction Models, One-Class Classification Models and Information-theoretic Models. The methods for anomaly detection can be further categorized into shallow and deep learning methods as surveyed in [4]. Recently, deep neural networks (DNNs) have shown a great capability to extract meaningful patterns from raw data with multiple levels of abstraction, providing state of the art results in various application fields like image recognition, object detection, speech recognition and natural language processing [5]. For unsupervised learning, approaches based on the Autoencoder (AE) framework [6] and Generative Adversarial Networks (GANs) [7] have proven helpful for anomaly detection. GANs are trained by employing a minimax game where a discriminator is trained to distinguish between real and fake data generated by a generator network. However, the training objective resulting in a saddle point convergence renders GANs notoriously hard to train. The AE framework encodes the multivariate sensor signal into a latent variable space by means of a DNN from which a decoder network reconstructs the input. AE architectures can be distinguished based on the form of input data corruption, and latent variable sampling they possess, namely Denoising AE [8], Variational AE (VAE) [9] and Adversarial AE [10]. In all approaches, the latent variable space constitutes an abstract representation of the input signals, which can infer between normal and abnormal conditions. However, as the training objective of the AE is the reconstruction loss between input and output, the discriminative power of latent variables to distinguish between operation modes is not enforced, which can result in poor performance in anomaly detection.

This paper tackles this problem and proposes a novel approach for anomaly detection in industrial processes based on a clustering-loss augmented convolutional autoencoder (CAE). We use a 1-dimensional CAE as the backbone architecture for the multivariate time-series task. In contrast to existing approaches, we split the latent space of the CAE into two sets, namely discriminative and reconstructive latent variables, and add an auxiliary loss for the discriminative latent variables. The loss is defined in terms of the well known K-means [11,12] clustering loss, where the auxiliary loss from the K-means algorithm during training is sampled only for the Top-K latent variables based on the greatest cluster centre distance achieved in clustering space. The reconstruction and the auxiliary loss are propagated through the discriminative latent variables, allowing for more discriminative hidden representation. We provide thorough experiments with unsupervised and semi-supervised approaches on the Tennessee Eastman [13] benchmark data set for anomaly detection. The results underline the applicability of the approach resulting in state-of-the-art performance.

The contributions of the paper can be summarized as follows:

- We present a novel unsupervised learning approach based on 1-dimensional convolutional neural networks and deep autoencoder structure where we define an auxiliary loss to increase the expressiveness of the latent representation.
- The proposed Top-K Deep Convolutional Clustering algorithm (Top-K DCCA) is novel in that the encoder parameters are divided into clustering and reconstruction subsets with the help of the Top-K operator. After this division, the encoder parameters from the clustering part are updated with an auxiliary clustering loss.
- We experiment with pure unsupervised and semi-supervised learning evaluation of the proposed method and report remarkable improvement on the Tennessee Eastman benchmark data set for anomaly detection. The results show the superior performance of the approach compared to the state-of-the-art.

The paper is organized as follows. In Section 2, the related work is presented. In Section 3 we state the considered problem, followed by the theoretical background on Clustering and Convolutional AE in Section 4. Section 5 presents the proposed approach for Convolutional Clustering-based unsupervised anomaly detection. In Section 6 we provide results and comparisons on the well-known Tennessee Eastman benchmark dataset. Section 7 concludes the paper.

## 2. Related Work

We discuss related work on data-based condition monitoring and anomaly detection in multivariate time series data and unsupervised and self-supervised learning approaches. Anomaly detection has been researched in various application fields and datasets. Some examples of datasets include the ISCX dataset [14] dataset for network intrusion detection, credit card fraud detection from the Mellon Bank Fraud Detection Feasibility Study [15] and health deterioration detection from the Oxford Cancer Hospital dataset [16]. The Tennessee Eastman dataset is chosen because the focus of this study is data-based condition monitoring for industrial processes.

Data-Based Condition Monitoring and Anomaly Detection: Condition monitoring and anomaly detection have a long history in various application domains. Anomaly detection for a production process can be seen as a sub-category in the condition monitoring field. In general, we can distinguish the existing works for condition monitoring in shallow learning and deep learning approaches. The various shallow learning approaches have been surveyed in [17,18,19]. Some examples of shallow methods for anomaly detection with unsupervised learning include Kernel Density Estimation [20], Principal Component Analysis [21], k nearest neighbours [22] and One-Class Support Vector Machines [23]. However, most of the mentioned shallow approaches are static, such that they cannot be efficiently used for time-series anomaly detection tasks. Additionally, extraction of relevant features from multivariate raw data is still a challenge with shallow methods. Deep domain knowledge of the process is required for choosing suitable techniques for feature extraction in the shallow approaches.

Different approaches from the deep learning field appear as high performing and efficient algorithms for condition monitoring and time series analysis. The deep learning architectures use multiple layers of non-linear transformations to extract high-level features from raw data, which provide relevant information for the respective task. The various deep learning approaches for condition monitoring have been surveyed in [24,25,26,27]. Most deep learning approaches consider supervised learning problems where faulty operation modes or process anomalies are labelled. However, the assumption of labelled data sets in industrial applications is too restrictive in various applications, including condition monitoring, remaining useful lifetime estimation and tool wear detection, to name a few. Hence, recent approaches also consider unsupervised deep learning approaches for anomaly detection and condition monitoring. Notably, deep AE and GANs have shown to be of particular use for such applications.

Deep Semi-supervised and Unsupervised learning: Deep learning models for anomaly detection have been used in various domains such as Intrusion Detection, Fraud detection, Malware detection, Medical detection [28]. We will highlight some specific examples from GANs and AE here. Anomaly detection for imaging markers relevant for disease progression with unsupervised learning based GANs has been reported in [29]. A semi-supervised learning based GAN has been presented for anomaly detection in multiple image datasets in [30]. Recently, there have been studies that use GAN for unsupervised fault diagnosis in rolling bearing [31] and semi-supervised fault diagnosis in planetary gearbox in [32]. Although recent improvements have been made in the GAN architecture, GANs are still known to have unstable training progress [33].

Deep AE, on the other hand, started the deep learning era in [34] and have been widely tested in various domains of anomaly detection such as brain scans [35], outlier detection in videos [36] and multiple public datasets from the UCI machine learning repository [37]. Recently, an automatic thermography defects detection using a spatial and temporal segmentation model has been proposed in [38]. A sparse mixture of Gaussian decomposition algorithm for inductive thermography has been proposed in [39]. Although deep AE for anomaly detection can be used in a supervised setting [40], we will focus on the methods for unsupervised and semi-supervised settings in production processes. An unsupervised learning based, memory augmented AE architecture has been proposed in [41] to better identify anomalies from normal data. A deep support vector data description method inspired by kernel-based one-class classification method for anomaly detection has been proposed in [42]. Stacked Sparse AE in a semi-supervised setting has been proposed in [43] for fault diagnosis in rotating machinery such as gearboxes. A similar semi-supervised learning approach for induction motor fault detection has been proposed in [44]. Unsupervised learning-based wind turbine monitoring with deep AE has been proposed in [45,46]. Unsupervised learning based spatiotemporal feature extraction methodology using Restricted Boltzmann Machines for fault detection has been proposed in [47]. Unsupervised Process monitoring with the variant AE has been presented in [48]. A comparison of deep AE, deep Denoising AE and VAE for semi-supervised anomaly detection approach in the TE process has been proposed in [49]. However, all of the previous methods are static approaches, which do not consider the dynamic nature of time-series data.

For time-series based anomaly detection, a Long Short Term Memory (LSTM) based encoder-decoder architecture has been proposed in [50]. Convolutional AE (CAE) was first presented in [51] for a higher level of feature extraction in images. CAE has, after that, been used for anomaly detection in images [52] and videos [53]. The Attention augmented Convolutional LSTM model has been proposed in [54] for anomaly detection in multivariate time series data. However, none of these approaches enhances the discriminative ability of the latent representation of the CAE model.

Deep Clustering: Some approaches in the literature join the use of feature extraction and clustering together to have better discriminative features. [55] proposed a joint clustering and reconstruction approach for image and text data. The main idea is to connect a clustering module at the bottleneck layer of an AE and optimize the parameters of the AE and the cluster centres jointly. A similar approach with CAE and clustering has been proposed in [56] for image data. Deep clustering has been also used for learning the weights of a convolutional network by using the cluster assignments as supervision [57]. Apart from K-means, an approach with KL-divergence minimization has been proposed in [58].

Our approach differentiates from the previous methods in two ways. First, we propose a Top-Kclustering approach where the latent space is divided into clustering friendly and reconstruction friendly spaces. Therefore, the latent features for reconstruction only get a gradient from the reconstruction error. However, the clustering features receive the update gradient from reconstruction and the clustering errors. Secondly, we apply the proposed approach on a multivariate time-series dataset from an industrial benchmark for anomaly detection. Therefore, the application field is very different from the usual image datasets.

## 3. Problem Statement

The main challenge for anomaly detection is to distinguish anomalous behaviour from data set noise. We conjecture that an incipient anomaly cannot be detected by one instance of the data set; instead, a specific time window of the input data set is required. Therefore, we concentrate on the analysis of multivariate time series data, i.e., we consider a sequence $\left\{x_{1},x_{2},\ldots ,x_{T}\right\}$ where $x_{i}\in R^{m}$ as input for the anomaly detection task, with *m* denoting the number of variables and *T* the length of the time-series signal. Further, we consider a hybrid, reconstruction-clustering based unsupervised learning methodology for anomaly detection, i.e., we assume that the evaluated data set is unlabeled. No indication is available whether the sequence exhibits normal or abnormal behaviour. Note, however, that for semi-supervised evaluation of the proposed approach, we use the learned AE for anomaly detection; labelled data is partly required and assumed to be known.

Then we can state the considered problem as follows: The purpose of the approach is to train the CAE structure $f_{\theta }\left(x\right)$, in such a way that the learned latent representation *z*, is able to best discriminate between normal $z_{no}$ and anomalous behavior $z_{ano}$, i.e., $|z_{no}-z_{ano}|\to \max$. Particularly, we aim to find an optimal separation between normal and anomalous data using unlabelled data only.

We present the solution that combines a deep CAE architecture with a latent representation clustering algorithm to find better discriminative latent representations.

## 4. Theoretical Background

### 4.1. K-Means Clustering

Clustering is one of the most profound and fundamental tasks in the field of unsupervised learning. However, various sets of factors make clustering notoriously complex. Some of these factors include [59]

- amount of noise in the data which can occur during data acquisition,
- use of data pre-processing techniques such as any form of dimensionality reduction,
- the clustering criterion and optimization algorithm is chosen and
- the initialization of the cluster centres.

These factors can affect the outcome of the clustering algorithm and can produce trivial solutions.

We keep the focus of our study to the K-means [11] algorithm. K-means, like most other data clustering algorithms, partitions the data into a pre-specified number of clusters. Clustering algorithms achieve this by minimizing a well-defined cost function involving the data and the assignment of the centres for each data instance. K-means belongs to the hard type, where each data point belongs to only one partition.

Formally, the task of clustering is to group *N* data samples into *K* clusters given a set of data samples ${\left\{x_{i}\right\}}_{i=1,\ldots ,N}$ where $x_{i}\in \;R^{M}$. The K-Means clustering algorithm achieves this goal by the optimization of the following cost function:

(1) $$\underset{M\;\in \;R^{M\;\times \;K},\;\left\{s_{i}\right\}\;\in \;R^{K}}{\mathrm{minimize}}\;\sum_{i=1}^{N}\|x_{i}-Ms_{i}{\|}_{2}^{2}$$

$$s.t.\;s_{j,i}\in \left\{0,1\right\},\;1^{T}\;s_{i}=1\;\forall i,j$$

where $s_{i}$ is the assignment vector of the *i*th data instance which consists of only one non-zero element, $s_{j,i}$ stands for the *j*th element of $s_{i}$, and the *k*th column of *M* stands for the centroid of the *k*th cluster.

The efficiency of the K-Means algorithm is the most when the data samples are evenly scattered around their centroids in their feature space. The data sets which possess this characteristic are called K-Means friendly data sets. However, this phenomenon rarely holds up in real-world data sets, because most of the real-world data sets are very high dimensional. Adding to that, most of the real-world data sets contain unwanted noise in the data. All these factors hinder the possibility of a data set being K-Means friendly [55].

To avoid these issues, usually, some form of dimensionality reduction or non-linear representation technique is used on the data set before applying K-Means. The K-Means algorithm applied to this non-linear representation usually yields better results [60]. The several available dimensionality reductions or non-linear representation techniques use Deep Neural Networks to learn better features from the data set. These methods are widely used for data pre-processing before applying K-Means, or other clustering algorithms [61].

### 4.2. 1-D CNN Autoencoder

**Figure 1:**
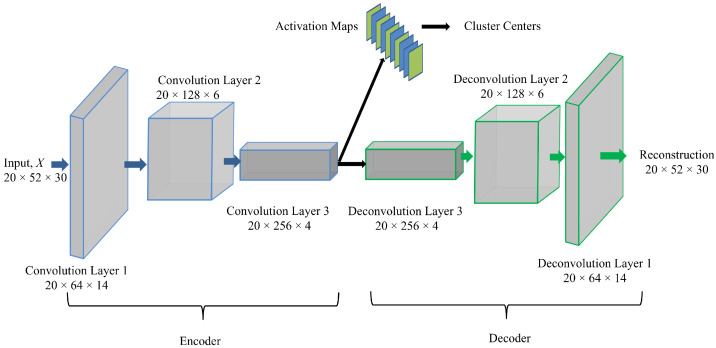
Proposed architecture of the clustering augmented deep autoencoder for anomaly detection.

The proposed encoder-decoder network architecture for the Top-K DCCA is shown in Figure 1, in which the encoder consists of 3 convolution layers, and the decoder comprises 3 deconvolution layers. Addtionally, there is a clustering module on the bottleneck representation of the encoder. The autoencoder applies a stack of 1-dimensional convolutional layers at both encoder and deconvolution layers at the decoder. The encoder transforms the multivariate time series data set to a latent representation thereby extracting relevant features of the data set. The decoder subsequently reconstructs the original data set from the general low dimensional latent representation. Since the decoder reconstructs the input based on the encoded representation of the bottleneck layer, i.e., Conv 3 layer, the activation maps from the Conv 3 layer can be considered as an encoded representation for a batch of the input dataset. Therefore, it is clear that the encoded representation has a verifiable relationship to the input features since the decoder recreates the input features from the activation maps in the encoded representation. The input size of each of the layers follows the naming convention as (Batch−Size×Number−of−Input−Channels×Sequence−Length).

On top of the latent representation, we employ a clustering module to make the latent representation more discriminative, allowing us better to capture the differences between normal and anomalous behaviour. As shown, we only employ the clustering on a subset of latent representations chosen based on different criteria to be discussed below. The rationale behind that architectural choice is to find a trade-off between consistent latent representations resulting in good reconstruction accuracy while making a subset of latent representation more discriminative, which suits downstream processing. In the following, we discuss the architectural modules in detail.

The combination of autoencoder structures with CNNs is a standard approach for deep unsupervised learning in various image and video processing tasks [53]. Here, at the encoder and decoder, convolutional and deconvolutional layers are employed to extract essential information within the latent representation. We use a similar approach to the time series analysis as proposed in [62], where the sensor channel and time dimensions make up the input to the network. As mentioned in the study, applying the standard 2-dimensional kernel is not appropriate as a meaningful relation between sensor channels is missing, resulting in poor performance. The 1D convolution operation is performed over a part of the complete input space, which is referred to as the receptive field. We denote the receptive field of size $n_{r}\times m$, which strides over the input T×m sequences, accounting for each of the variables. The *p*th convolution 1D kernel in the first layer can be denoted with a 2-dimensional tensor $K^{\left(p\right)}=\left[k_{i,j}^{\left(p\right)}\right]\in R^{n_{r}\times m}$. The indices i,j denote the dimension along the time and variable axis, respectively. The outputs or feature maps extracted from the convolution operation with 1 convolution kernel is a 1-dimensional tensor $H=[h_{i}]$. Usually, multiple convolution kernels are used in each convolution layer leading to multiple feature maps, which subsequently make the feature maps a 2-dimensional tensor $H=[h_{i,p}]$. Each convolution kernel is responsible for extracting different features from the input data. Formally, the convolution 1D operation can be summarized as follows:

(2) $$\begin{array}{c} h_{i,p}={\left(x*k\right)}_{i}=\sum_{g=1}^{n_{r}}\sum_{f=1}^{m}x_{i+g-1,f}\cdot k_{g,f}^{p}\\ \forall i\in \{1,\ldots ,T-n_{r}+1\}\\ \forall p\in \{1,\ldots ,d_{q+1}\}, \end{array}$$

where $h_{i,p}$ denotes the output of the ${\left(i\right)}^{th}$ receptive field and the *p*th convolution kernel, $x_{i+g-1,f}$ are the elements in the receptive field of the input variable, $k_{g,f}$ is the convolution kernel and $d_{q+1}$ denotes the number of convolution kernels in the given layer.

The deconvolution, sometimes called the transposed convolution operation, performs the inverse operation as the convolution operation, such that it up-samples the individual feature maps into the original input. The weights of the convolution and deconvolution filters can be tied, but we keep them untied in this study.

As we cope with time series of variable length where the time dimension is significant, we employ a sliding window approach for the time dimension. As such, we define a window of size of $m_{w}\times n$ with $T>>m_{w}>m$, which is analyzed within one processing step of the deep autoencoder. Then the time series is strided in the time dimension by a stride of $s_{w}$ to define a new window to be processed in the next step. This approach has some advantages compared to processing directly on the complete input sequence. Notably, an individual data point $\left\{x_{i}\right\}$ is processed more than once in different settings, increasing the robustness of the resulting convolution kernels.

## 5. Convolution Clustering Based Unsupervised Learning for Anomaly Detection

In this section, we propose the training strategy for the unsupervised learning approach for the Top-K DCCA approach.

### 5.1. Top-K DCCA

We augment the previously defined CAE architecture by a novel Top-Kclustering objective defined on a subset of the latent space as illustrated in Figure 1. Particularly, we split the latent space into two subsets of latent variables $Z_{c}\subseteq R^{n_{c}}$ and $Z_{r}\in R^{n_{rec}}$ which we term clustering and reconstruction friendly latent variables in the following. The rationale behind the split of the latent space is to better weigh-off between reconstruction accuracy and discriminative clustering accuracy. Hence, we force consistent representation of the input data by the reconstruction space and the discriminative power of the clustering features to improve performance on downstream tasks.

As such, the clustering related latent variables are passed through an arbitrary clustering algorithm. We employ the well-known K-means algorithm for clustering in this work due to its simplicity. However, we emphasize that various other clustering approaches can be combined with our framework. The k-means algorithm is subsequently used on the latent representation Z, leading to the optimization of the following cost function:

(3) $$\begin{array}{c} \min_{M_{j}\in R^{n_{c}\times k},s_{i}\in {\left\{0,1\right\}}^{K}}\sum_{i=1}^{N}\|z_{j}^{i}-M_{j}s_{i}\| \end{array}$$

(4) $$\begin{array}{c} s.t.\;1^{T}s_{i}=1\;\forall i,\; \end{array}$$

(5) $$\begin{array}{c} \forall z_{j}\in Z, \end{array}$$

where the column vector $m_{k,j}$ of *M* denotes the *k*th cluster center in the $n_{c}$-dimensional space and $s_{i}$ is the cluster assignment of the *i*th data points latent representation.

A crucial part of the system setup is the split of the latent space. A straightforward approach would be to separate cluster and reconstruction friendly latent variables before training. However, this appears to be restrictive when used together with the CAE, particularly during training. Hence, instead of defining the split at the start of training, we augment the K-means clustering by a Top-K sampling method that uses the top-$n_{c}$ latent variables in terms of their discriminative performance. The splitting criterion is the euclidean distance between the 2 cluster centers present in each of the latent variables. The Top-K operation of latent variables ranking returns indices of the K latent variables where the distance between the cluster centers is maximum. The discriminative performance is measured based on the euclidean distance between the cluster centres in the latent space. According to the authors, the maximum distance between the cluster signifies that the latent variable has more discriminative performance since it can efficiently identify the 2 different operating conditions. Specifically, if we assume an anomaly detection task with two clusters with centres $m_{no,j}$ and $m_{ano,j}$ indicating normal and anomalous operation, respectively, we employ the following euclidean distance measure

(6) $$\begin{array}{c} \max_{j\in Z_{c}}d\left(m_{no,j},m_{ano,j}\right)=\max_{j\in Z_{c}}\|m_{no,j}-m_{ano,j}{\|}^{2}, \end{array}$$

to identify the Top-$n_{c}$ latent variables forming the set $Z_{c}$.

It is important to note that the clustering loss is employed independently on the latent variables in the set $Z_{c}$. However, during training, we fed back the loss of the top-$n_{c}$ latent variables only. Therefore, during training, the latent variables switch among the clustering subset and reconstruction subset, based on the euclidean distance of their respective cluster centers. This ensures that a subset of latent space is discriminative by forcing the model to learn a hidden representation in which certain cluster centers are as far away as possible based on the criterion from Equation (6). During the testing phase, the trained division latent space into the 2 subsets is kept constant.

The split percentage of the latent variables defined by $n_{c}$, $n_{r}$ is a hyperparameter that has to be determined a priori. It has to trade-off between reconstruction and discrimination capability of the latent variable space. In practice, we found a 50/50 split between working well in all the experiments.

### 5.2. End-to-End Training of the Clustering Augmented AE

This section introduces the end-to-end training for the clustering augmented deep autoencoder. Particularly, we discuss the interaction between the loss propagation of the clustering and the reconstruction module of the autoencoder. The parameters of the CNN of both encoder $f_{\theta }$ and decoder $g_{\psi }$ are trained by the reconstruction loss between input and reconstructed output, i.e.,

(7) $$\begin{array}{c} L_{\mathrm{AE}}\left(\theta ,\psi \right)=\sum_{i=1}^{N_{B}}\|x^{i}-g_{\psi }\left(f_{\theta }\left(x^{i}\right)\right){\|}_{2}^{2}, \end{array}$$

where $N_{B}$ is the minibatch size. Additionally, we feed back the clustering loss through the clustering friendly latent variables

(8) $$\begin{array}{cc} L_{j,\mathrm{CL}}\left(\theta \right) & =\sum_{i=1}^{N_{B}}\|z_{j}^{i}-M_{j}s^{i}{\|}_{2}^{2}=\sum_{i=1}^{N_{B}}\|f_{j,\theta }\left(x^{i}\right)-M_{j}s^{i}{\|}_{2}^{2}, \end{array}$$

(9) $$\begin{array}{cc} z_{j} & \in Z_{c}, \end{array}$$

which subsequently affect the encoder parameters only.

**Figure 2:**
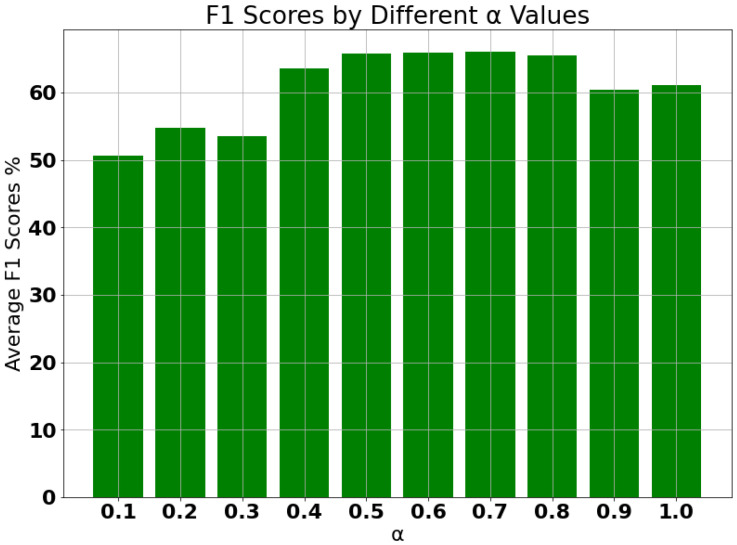
Average $F_{1}$ score of the model on all the fault cases based on different values of α.

The total loss for training the CAE is

(10) $$L\;=\;\alpha \sum_{j=1}^{z_{j}}L_{j,\mathrm{CL}}\left(\theta \right)\;+\;\left(1\;-\;\alpha \right)L_{\mathrm{AE}}\left(\theta ,\psi \right)$$

where the value of α ranges between 0.6 to 1, and it acts as a weighing factor between the two loss functions. This range of optimal value of α was empirically found based on the average $F_{1}$ score that was achieved on all the fault cases. The experimental results on the different values of α are illustrated in Figure 2.

It is considered an additional hyperparameter of the network and has to be tuned while training it. Since α≤1 keeps the overall loss distribution towards the reconstruction and clustering losses balanced.

It is theoretically possible to chose a a different independent parameter β, with the condition that α+β=1. However, to keep the number of hyperparameters in check, this setting of just one hyperparameter α has been chosen.

The gradient of the above equation with respect to the network parameters can be computed from the equation below:

(11) $$\nabla \;\chi \;L=\left(1\;-\;\alpha \right)\;\frac{\partial L_{\mathrm{AE}}}{\partial \chi }\;+\;\alpha \sum_{j=1}^{z_{j}}\frac{\partial L_{j,\mathrm{CL}}}{\partial \theta }$$

(12) $$\nabla \;\chi \;L=\left(1\;-\;\alpha \right)\;\sum_{i=1}^{N_{B}}2(x^{i}-g_{\psi }\left(f_{\theta }\left(x^{i}\right)\right){\left[g_{\psi }\left(f_{\theta }\left(x^{i}\right)\right)\right]}^{\prime }\;+\;\alpha \sum_{i=1}^{N_{B}}\sum_{j=1}^{z_{j}}2\left(f_{j,\theta }\left(x^{i}\right)-M_{j}s^{i}\right)f_{j,\theta }{\left(x^{i}\right)}^{\prime }$$

where χ=(θ,ψ) is the collection of encoder and decoder parameters and the partial gradients are calculated by back-propagation [63]. Subsequently, the network parameters are updated with gradient descent as

(13) χ←χ−β∇χL

where β is the learning rate.

During the initial stages of training, termed as pre-training, the value of α is set to 0. This ensures that the network learns from only the reconstruction loss. Since no clustering loss is imposed on the network, the network tries to reconstruct the input solely based on the non-clustering loss. For the clustering augmented training stage, a fixed value of α is set. The network is trained on both loss functions. This method ensures that the reconstruction of the input is taken into account and helps to avoid trivial solutions. In addition, we define a Cluster Update Interval *C*, which denotes the interval in which the cluster centres of the latent feature representation are updated to have robust hidden representation.

The algorithm of the Top-K DCCA is represented in Algorithm 1, where a model is trained for *N* epochs.
**Algorithm** **1** Top-K Deep Convolutional Clustering Algorithm.  1: **procedure**
Initialization (Perform N epochs over the data)  2:       P = Number of pre-training epochs  3:       C = Cluster update interval  4:       **for** epoch = 1 to P + 1 **do**  5:             Reconstruct the data, extract latent representation $f_{\theta }\left(x^{i}\right)$  6:             Compute gradients $\nabla \;\chi \;L^{i}$ with α=0 by Equation (11)  7:             Update network parameters χ by Equation (13)  8:             **if** epoch = P + 1 **then**  9:                   Perform K-Means optimising the Equation (3)10:                   Return centers $m_{no,j}$ and $m_{ano,j}$ and center assignments $M_{j}s_{i}$11:                   Rank latent representation layer channels by Equation (6)12:                   Return Top *K* ranked channels13:       **for** epoch = P + 1 to N **do**14:             Reconstruct the data, extract latent representation $f_{\theta }\left(x^{i}\right)$15:             Compute gradients $\nabla \;\chi \;L^{i}$ with α=0 by Equation (11)16:             Update top *K* ranked channel parameters by Equation (13)17:             Zero the gradients18:             Compute gradients $\nabla \;\chi \;L^{i}$ with α=0 by Equation (11)19:             Update rest of the channel parameters by Equation (13)20:             **if** epoch % C = 0 **then**21:                   Perform K-Means by optimising the Equation (3)22:                   Return centers $m_{no,j}$ and $m_{ano,j}$ and center assignments $M_{j}s_{i}$23:                   Rank latent representation layer channels by Equation (6)24:                   Return Top *K* ranked channels

## 6. Experimental Results

### 6.1. Tennesse Eastman Benchmark

The TE process was originally created by Downs and Vogel as a process control challenge problem in [13]. The generated dataset from the TE Process consists of 22 continuous process measurements, 19 component analysis measurements, and 12 manipulated variables. The dataset consists of 21 pre-programmed faults, among which 16 are known fault cases, and 5 fault cases are unknown. Both the training and testing datasets include a total of 52 observed variables. The training dataset consists of 22 different simulation runs, and simulation 0 is fault-free. In our case, this simulation is considered as our normal data sample. Simulations 1 to 21 were generated for 21 fault cases, and in our case, all of these 21 simulations are considered anomalous data samples. Similarly, the testing data set contains 22 different simulations, the first one being the normal case, and the rest are simulations for different fault cases. Table 1 represents the Tennessee Eastman Process fault cases. Since the TE process dataset contains collected time-series sensor data, the data is prepared as time series sequences as discussed in [1] before the training.

### 6.2. Training Setup

The length of each sequence is decided prior to the training, and both the data with and without faults are arranged into time-series sequences. This kind of arrangement has proved to help the model in increasing the performance since a time-series gives more context about the situation than a single measurement. We select a sequence length of 30 for our experiments as this length gives a good overall performance.

To define the anomaly detection setting, we follow previous works [1] by dividing the fault classes into subgroups based on how challenging the faults are to detect. Accordingly, we divide the 21 faults into three subgroups: easy, medium, and hard-to-detect faults. The three fault subgroups considered are as shown in Table 2. The data from the literature have been adapted accordingly for comparison.

For evaluation of the anomaly detection task, we concentrate on measures related to the numbers of correctly and incorrectly classified data points. Specifically, we use the standard notions of true positives (TP) and true negatives (TN) to denote the number of examples predicted correctly as a positive and negative class, respectively and false positives (FP) and false negatives (FN) as the number of examples predicted incorrectly as a positive and negative class, respectively. Based on the values, we use the $F_{1}$ score as the performance measure. The $F_{1}$ score is chosen as the evaluation metric because if the number of examples in one of the classes is higher than the other, then even random guessing can result in high prediction accuracy. Therefore, we use the $F_{1}$ score, which is a geometric mean of precision *P* and recall *R*, is considered in the case of the TE process given as

(14) $$\begin{array}{c} F_{1}=2\frac{P\cdot R}{P+R}, \end{array}$$

where

(15) $$\begin{array}{cc} P & =\frac{TP}{TP+FP}, \end{array}$$

(16) $$\begin{array}{cc} R & =\frac{TP}{TP+FN}. \end{array}$$

We apply the proposed learning methodology to the TE benchmark data set and provide a thorough ablation study. The comparison study is enlisted as follows.

- We start by comparing the fault detection capabilities for completely unsupervised learning techniques in which the proposed methodology is compared to the standard k-means augmented CNN approach.
- We then evaluate the fault detection capabilities with semi-supervised learning techniques, in which the proposed methodology is pre-trained with unlabelled data and finally, a fully connected layer is fine-tuned with labelled data. This technique is compared with and without K-means clustering, with and without Top-K K-means clustering.

In this section, we defined the training setup for the anomaly detection task on the TE process. Based on this setup, experimental results and ablation studies were performed to evaluate the prediction performance of the proposed methodology.

### 6.3. Unsupervised Learning Results

This section presents the results obtained by applying the proposed approach Top-K DCCA in a purely unsupervised learning setting. This means that no labels from the fault information have been used for training the models. The results obtained from the proposed approach are compared with the baseline architecture, hereafter referred to as the Vanilla architecture, and a standard DCCA approach. The Vanilla architecture is a 3 convolution layer architecture, whereas the Top-K DCCA model is tested with a 2 and 3 layer convolution layer architecture. The architecture description for the Vanilla, DCCA and the Top-K DCCA architecture is as follows:

- Three convolution layers with the LeakyReLU [64] activation function
- A kernel size of 3 in all convolution layers
- The number of convolution channels doubling with each layer, starting with 64 channels.
- The number of clustering channels is set to 128 in the bottleneck layer.
- A batch-size of 20 with α=0.6 and β=0.001 is used.
- All the models are trained for 100 epochs with the stochastic gradient descent (SGD) optimizer with an L2 penalty of 0.02.

Anomaly detection in the Vanilla architecture is obtained by performing K-means clustering once after the training process, whereas in the other two architectures, K-means clustering is part of the training process.

**Figure 3:**
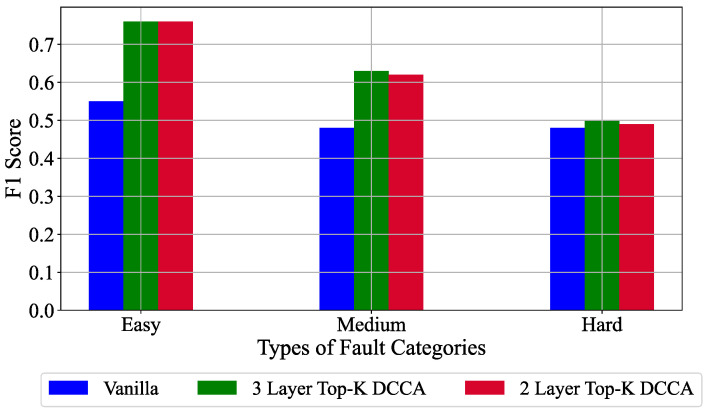
$F_{1}$ score obtained by the Vanilla and the Top-K DCCA approach with different layers for anomaly detection task in an unsupervised learning setup.

To evaluate the prediction performance of the proposed architecture, a 2 and 3 layer Top-K DCCA architecture is compared to the Vanilla model for the anomaly detection task in the TE process. The prediction performance in terms of $F_{1}$ score for the best performing architectures is shown in Figure 3. It is clear from Figure 3 that the proposed architecture performs drastically better than the baseline model on all the fault categories in the 2 layer and the 3 layer configuration. The 3 layer configuration performs slightly better than the 2 layer one in all the cases. Therefore, for the subsequent analysis, we keep the 3 layer configuration.

**Figure 4:**
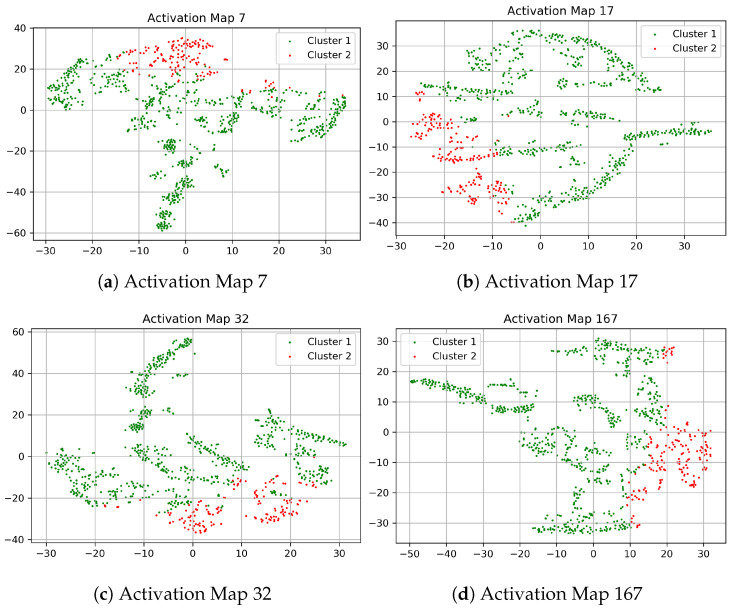
t-SNE Visualization of a sample of the activation maps with Top-K DCCA Approach on Tennessee Eastman Data.

To better visualize the discriminative capability in the latent representation, the t-SNE [65] plots of some of the clustering friendly activation maps are shown in Figure 4. In all of these t-SNE visualizations of the activation maps, the model has learned through the training process that there are two distinct regions, i.e., normal and anomalous regions. The Figure 4 show two clusters because the Tennessee Eastman process dataset consists of either normal operation or faulty operation. That is why we limit the number of clusters to just two. The boundaries of the two distinct regions can be clearly seen, which demonstrates that the clustering operation has helped create these decision boundaries. The t-SNE visualizations show the distinct separation for most of the test samples. Some of the data samples from the two operating conditions are close to each other, signifying the hard to detect anomaly samples.

The unsupervised training results and the corresponding t-SNE plots prove the applicability of the proposed methodology to effectively identify anomalies in a dynamic and high-dimensional time-series process. A 3 layer unsupervised learning based Top-K DCCA approach performs the best under the considered experimental settings.

### 6.4. Semi-Supervised Learning Results

**Figure 5:**
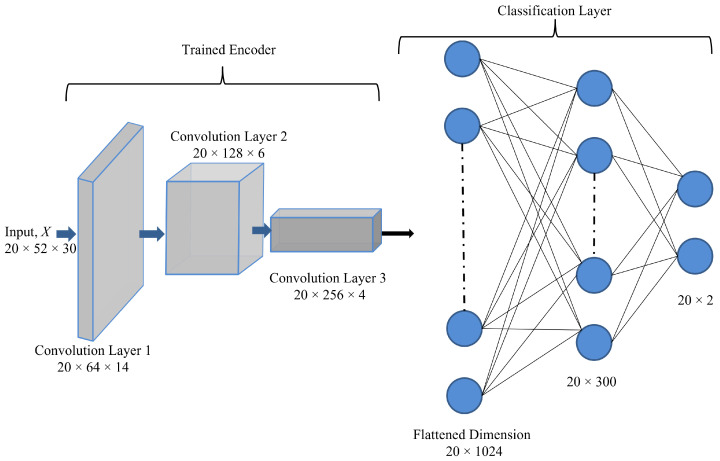
Proposed architecture for semi-supervised deep autoencoder for anomaly detection.

In this section, we present the results from the semi-supervised training setup where the encoder of the Top-K DCCA architecture is pre-trained with unlabelled data as per Algorithm 1, with two fully connected layers with 300 and 2 hidden units being trained in a supervised manner with labelled data. The overall proposed architecture for semi-supervised learning is shown in Figure 5. The convolutional encoder is pre-trained using unlabelled data and the fully connected layers are fine-tuned using labelled data. During the fine-tuning stage, the weights and biases of the convolutional encoder are frozen.

**Figure 6:**
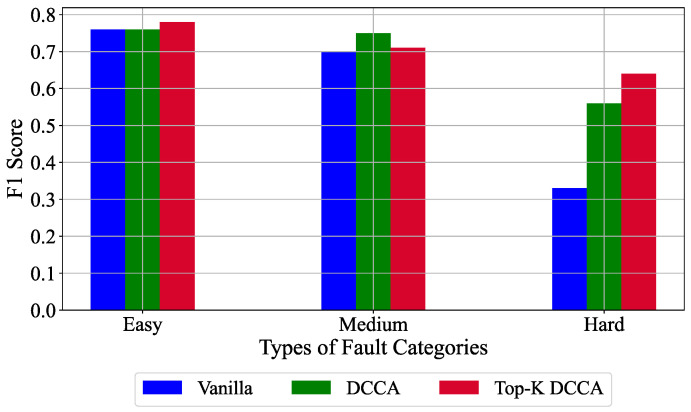
$F_{1}$ score obtained by the Vanilla, DCCA and the Top-K DCCA approach for the anomaly detection task in a semi-supervised learning setup.

**Figure 7:**
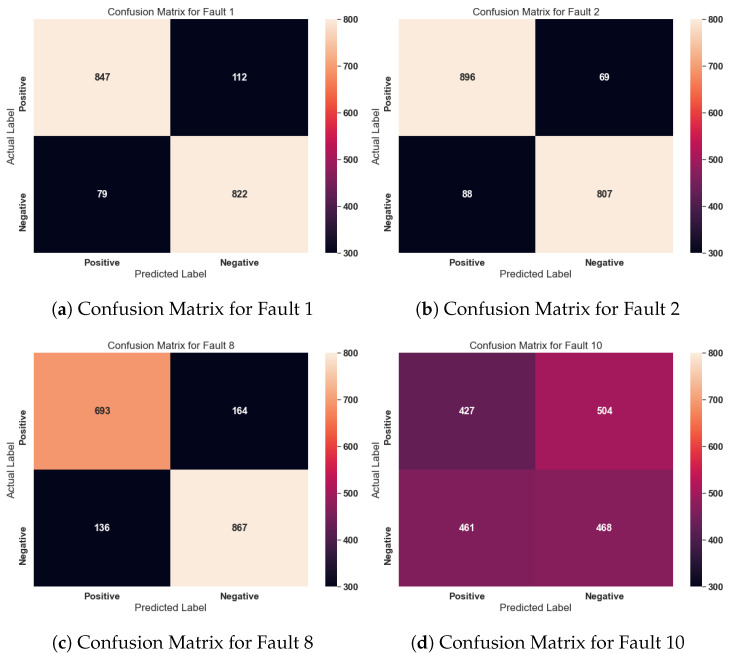

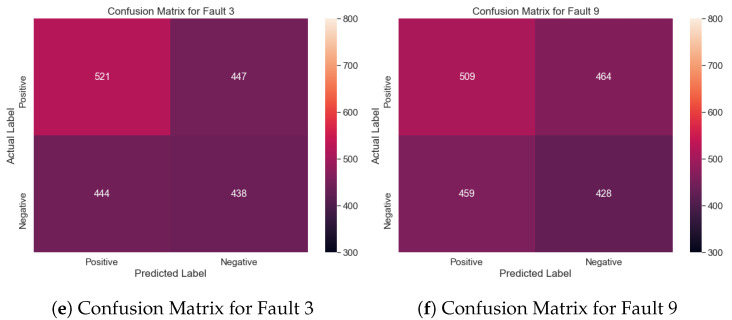
Confusion matrix for a sample of fault cases from the Easy, Medium and Hard fault groups. The positive class represents normal case and the negative class represents the respective fault case.

The average $F_{1}$ score obtained by the Vanilla, DCCA and Top-K DCCA approach on the different fault categories is shown in Figure 6. It is clear from Figure 6 that the proposed Top-K DCCA approach outperforms the other two models in the Easy and Hard fault categories drastically. The standard DCCA only marginally performs better in the medium category; however, the proposed methodology works better than the Vanilla model in all three fault categories. To better estimate the anomaly detection performance of the model, confusion matrices for a sample of fault cases from the Easy, Medium and Hard fault groups have been illustrated in Figure 7. The confusion matrix from all the fault cases has not been added for the brevity of results. The confusion matrix for fault 1 and fault 2 shows that the model can distinguish the normal and faulty cases in most cases. However, the model has difficulty distinguishing some medium and hard fault cases from the normal case. This can be observed from the low performance on fault cases 3, 9, and 10. It must be noted here that semi-supervised learning results are comparatively better than the unsupervised learning results since labelled data is used to train the final hidden layers.

### 6.5. Classification Variants Results

**Figure 8:**
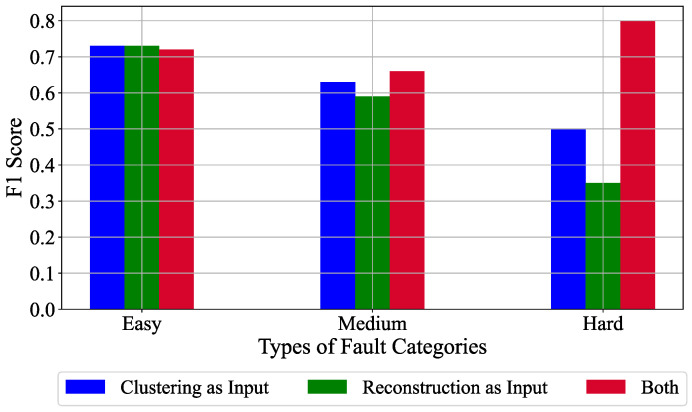
$F_{1}$ score obtained by the different classification variants for the anomaly detection task in a semi-supervised learning setup.

In this section, we present the results for the different classification variants that are possible with the proposed Top-K DCCA approach based on the semi-supervised learning approach. The classification variants include feeding only the clustering channels $Z_{c}$ as input, reconstruction channels $Z_{r}$ as input or both the sets together to the two fully connected layers. The architecture for the classification remains the same as in Figure 5. These different classification variants are done to observe how much each of the latent variables sets help in the final anomaly detection task. The average $F_{1}$ scores obtained by the three classification variants on the different fault categories is shown in Figure 8. It is clear from Figure 8 that the clustering set of latent variables $Z_{c}$ as input performs consistently better than the reconstruction set $Z_{r}$ as an input across all the different fault categories. This result emphasizes the importance of the Top-K clustering channels in the anomaly detection task. It must be noted, however, that using both the sets as input to the fully connected layers also drastically helps in improving the performance in the case of Medium and Hard fault cases.

### 6.6. Comparison with Literature

In this section, we provide a comparison of the anomaly detection performance of the proposed Top-K DCCA model with other existing approaches. We emphasize the performance of the hard to detect fault cases since having a good performing model on these cases is a challenging task. Since most of the previous works use a percentage based evaluation metric, the $F_{1}$ score is multiplied by 100 to keep the comparison uniform. For the comparison, we selected the previous studies [21,66,67] and chose the best performing models Independent Component Analysis, Dynamic Principal Component Analysis with decorrelated residuals and canonical variate analysis, respectively. Furthermore, to compare the model’s with other deep learning models, the Deep Autoencoder (DAE) and Denoising DAE have been selected from the previous work in [49]. The Table 3 gives the comparison between the best performing unsupervised learning-based anomaly detection approaches with their achieved $F_{1}$ scores or fault detection rates as used in literature. The data from the literature have been adapted accordingly for comparison. The proposed Top-K DCCA model outperforms the existing literature methods in three out of the four fault cases and has a drastically better overall performance. In comparison to the other neural network approaches using fully connected layers, the proposed Top-K DCCA approach outperforms these methods on all hard to detect fault cases. The exceptional performance gain underlines the anomaly detection capability of the proposed model, especially in the case of incipient anomaly cases.

## 7. Conclusions

We presented a novel approach for unsupervised training of time series data sets with a particular focus on anomaly detection. The approach combines a deep 1D-CNN-based autoencoder with a clustering loss on a subset of the latent variable space, which increases the discriminative power within the latent variable space without sacrificing too much reconstruction performance on the data set. We make the approach end-to-end trainable by backpropagating both the clustering and the reconstruction objective through the network. We test the approach on the Tennessee Eastman benchmark data set with very encouraging results. In the unsupervised learning setting, a 3 layer proposed model drastically outperforms other deep Autoencoder networks and also shallow learning techniques proposed in the literature. The ablation studies in the semi-supervised learning setting show the superior performance of the model using the input from the clustering feature subset as compared to the reconstruction feature subset. This shows the discriminative power of the learnt features in the latent space.

In the future, authors would apply the proposed approach to other time-series datasets like Electric devices, Ford A and Ford B [68] to corroborate and confirm our findings.

## Figures and Tables

**Table 1:** Tennessee Eastman Process Fault Cases.

Fault Cases	Description	Type
1	A/C ratio, B composition constant (Stream 4)	Step
2	B composition, A/C ratio constant (Stream 4)	Step
3	D feed temperature (Stream 2)	Step
4	Reactor cooling water supply temperature	Step
5	Condenser cooling water supply temperature	Step
6	A feed loss (Stream 1)	Step
7	C header pressure loss (Stream 4)	Step
8	A, B, C feed composition (Stream 4)	Random
9	D feed temperature (Stream 2)	Random
10	C feed temperature (Stream 4)	Random
11	Reactor cooling water supply temperature	Random
12	Condenser cooling water supply temperature	Random
13	Reaction Kinetics	Slow drift
14	Reactor cooling water valve	Sticking
15	Condenser cooling water valve	Sticking
16	Unknown	-
17	Unknown	-
18	Unknown	-
19	Unknown	-
20	Unknown	-
21	A, B, C feed valve (Stream 4)	Constant position

**Table 2:** Fault Groups in TE Process [1].

Subgroup	Normal Case	Fault Cases
Easy	0	1, 2, 4, 5, 6, 7, 12, 14, 18
Medium	0	8, 10, 11, 13, 16, 17, 19, 20
Hard	0	3, 9, 15, 21

**Table 3:** Comparison of the achieved $F_{1}$ scores for the hard to detect fault cases with existing approaches.

Fault Case	Top-K DCCA	[21]	[66]	[67]	DAE [49]	Denoising DAE [49]
(3)	53.82	4.5	2.1	1.4	16.66	16.67
(9)	52.31	4.75	2	0.7	16.87	16.97
(15)	43.98	7.75	38.5	9.7	17.08	17.08
(21)	50.05	56.38	53.9	65.8	44.37	45
Overall	50.04	18.34	24.12	19.4	23.74	23.93

## Abbreviations

The following abbreviations are used in this manuscript:

DNN	Deep Neural Networks
CNN	Convolutional Neural Networks
AE	Autoencoder
CAE	Convolutional Autoencoder
VAE	Variational Autoencoder
GAN	Generative Adversarial Networks
TE	Tennessee Eastman
DCCA	Deep Convolutional Clustering Algorithm
LSTM	Long Short Term Memory
